# Fast peak error correction algorithms for proteoform identification using top-down tandem mass spectra

**DOI:** 10.1093/bioinformatics/btae149

**Published:** 2024-03-18

**Authors:** Zhaohui Zhan, Lusheng Wang

**Affiliations:** Department of Computer Science, City University of Hong Kong, Kowloon, Hong Kong, China; Department of Computer Science, City University of Hong Kong, Kowloon, Hong Kong, China; City University of Hong Kong Shenzhen Research Institution, ShenZhen, 518057, China

## Abstract

**Motivation:**

Proteoform identification is an important problem in proteomics. The main task is to find a modified protein that best fits the input spectrum. To overcome the combinatorial explosion of possible proteoforms, the proteoform mass graph and spectrum mass graph are used to represent the protein database and the spectrum, respectively. The problem becomes finding an optimal alignment between the proteoform mass graph and the spectrum mass graph. Peak error correction is an important issue for computing an optimal alignment between the two input mass graphs.

**Results:**

We propose a faster algorithm for the error correction alignment of spectrum mass graph and proteoform mass graph problem and produce a program package TopMGFast. The newly designed algorithms require less space and running time so that we are able to compute global optimal alignments for the two input mass graphs in a reasonable time. For the local alignment version, experiments show that the running time of the new algorithm is reduced by 2.5 times. For the global alignment version, experiments show that the maximum mass errors between any pair of matched nodes in the alignments obtained by our method are within a small range as designed, while the alignments produced by the state-of-the-art method, TopMG, have very large maximum mass errors for many cases. The obtained alignment sizes are roughly the same for both TopMG and TopMGFast. Of course, TopMGFast needs more running time than TopMG. Therefore, our new algorithm can obtain more reliable global alignments within a reasonable time. This is the first time that global optimal error correction alignments can be obtained using real datasets.

**Availability and implementation:**

The source code of the algorithm is available at https://github.com/Zeirdo/TopMGFast.

## 1 Introduction

Recent studies show that changes in protein isoform expression and post-translation modifications (PTMs) have gained recognition for their roles in underlying disease mechanisms (Brown *et al.* 2020). Besides, all protein products that arise from a single gene due to genetic variations, alternative splicing, and PTMs can significantly diversify the proteome for basic and clinical research (Melby *et al.* 2021). Proteoform identification is a challenging problem that has been attracting lots of attention.

Traditional ‘bottom-up’ mass spectrometry (MS)-based proteomics has become less practical for high-throughput proteoform identification due to the ‘peptide-to-protein’ inference problem with the low protein sequence coverage and a loss in proteoform information (Toby *et al.* 2016, Schaffer *et al.* 2019). For this reason, ‘top-down’ MS-based proteomics was coined by McLafferty and co-researchers in 1999. This ‘top-down’ MS-based proteomics analyzes intact proteins instead of digesting them into peptides, which enables accurate proteoform identification, PTM localization, and relative quantification for well understanding essential biological functions, unraveling disease mechanisms and discovering new biomarkers (Shaw *et al.* 2013, Riley *et al.* 2017, Schaffer *et al.* 2018, Dai *et al.* 2019, Arauz-Garofalo *et al.* 2021, Melby *et al.* 2021). Besides, when multiple PTMs coexist on single protein molecules, ‘top-down’ MS-based proteomics becomes the only feasible method for characterization (Holt *et al.* 2019).

For proteoform identification, we need to align a top-down spectrum against a protein database. To handle the combinatorial explosion of possible proteoforms, the proteoform mass graph was proposed (Kou *et al.* 2017), and a program package TopMG was produced. Moreover, a top-down spectrum can be represented as a spectrum mass graph. The problem of searching a spectrum against a database is formulated as finding an optimal alignment between the two mass graphs. Here we design new algorithms for aligning the two mass graphs with special treatment on spectrum peak error handling.

When aligning the peaks of the spectrum with proteoform mass graphs, the theoretical mass values on the proteoform mass graphs are not necessarily identical to the mass values of corresponding peaks since the mass values of peaks have errors. In previous methods, an error tolerance value δ is used to handle this issue and for any two consecutive nodes in the obtained alignment, the masses between the two consecutive nodes in the spectrum mass graph is within the range [x − δ,x + δ], where *x* is the theoretical mass between the two consecutive nodes in the proteoform mass graph. This method has a serious problem in that when having a global view of the whole obtained alignment, one may find that the mass between two nodes in one graph is significantly different from that of the two corresponding nodes in the other graph due to some consecutive positive/negative error accumulation. However, any big difference in masses on the two input mass graphs between any pair of matched nodes is clear evidence that the obtained alignment is not reliable. Thus, it is necessary to have some special ways to handle this issue.

Some heuristic methods have been used in Kou *et al.* (2017). A new model was proposed in Zhan and Wang (2022) to handle spectrum peak errors and the corresponding program package is referred to as TopMGRefine. They require that each matched node *y*${\;}_{i}$ (peak) with mass value *m*${\;}_{i}$ in the spectrum mass graph has an error correction value *k* so that $m_{i}\;+\;k$ is the ‘true mass’ after error correction, which should be the same as the theoretical mass in the proteoform mass graph. The problem is referred to as the *error correction alignment of spectrum mass graph and proteoform mass graph problem*. The dynamic programming algorithm given in Zhan and Wang (2022) needs to have an extra index *k* so that the running time of the algorithm is increased by a factor of *k* and it is extremely slow in practice. Thus, the authors can only provide a program that can compute a local optimal alignment of two input mass graphs with additional input information of the alignment starting nodes in the two graphs (Zhan and Wang 2022). They propose to use existing methods to provide a few candidate starting nodes and use their program to get better quality local alignments.

In this article, we propose a faster algorithm for the error correction alignment of spectrum mass graph and proteoform mass graph problem and produce a program package TopMGFast in C++. The newly designed algorithms require less space and running time so that we are able to compute global optimal alignments for the two input mass graphs in a reasonable time.

For the local alignment version, we used the 2817 protein and spectrum pairs obtained after filtering for experiments. Both TopMGRefine and TopMGFast obtain identical local alignment results. The total running time of the 2817 protein and spectrum pairs for TopMGrefine and TopMGFast is 4760 and 1715 min, respectively. That is, the new algorithm is much faster.

For the global alignment version, the same dataset is used. Since TopMGRefine does not support a global alignment version due to large memory and running time requirements, we compare our program with TopMG. Experiments show that the maximum mass errors between any pair of matched nodes in the alignments obtained by our method are within a small range as designed, while TopMG-generated alignments have very large maximum mass errors for many cases. The obtained alignment sizes are roughly the same for both TopMG and TopMGFast. Of course, TopMGFast needs more running time. In fact, the running time of TopMGFast is 3 times that of TopMG. Therefore, our new algorithm can obtain more reliable global alignments within a reasonable time. This is the first time that global optimal error correction alignments can be obtained using real datasets.

## 2 Materials and methods

To deal with all possible proteoforms of a protein, Kou *et al.* formulate a protein and all its possible proteoforms as a *proteoform mass graph* (PMG for short) (Kou *et al.* 2017). Each amino acid in a protein has a left node and a right node corresponding to the bonds left and right to the amino acid. An edge connecting the two nodes is assigned the mass of the amino acid. Each edge corresponding to an original residue has a black color. For each modification of an amino acid, there is a new edge with the modified mass connecting the two nodes of the original amino acid. The edges corresponding to modifications of amino acids have a red color. The PMG has a unique starting node and an ending node.

A spectrum is also formulated as a *spectrum mass graph* (SMG for short), where there is a special node *y_0_*, each peak *p*${\;}_{i}$ with mass *m*${\;}_{i}$ in the spectrum corresponds to a node *y*${\;}_{i}$ for i > 0 in the SMG, there is a directed edge connecting *y*${\;}_{i}$ and $y_{i+1}$ with mass $m_{i+1}-m_{i}$ such that the length of the path from *y_0_* to *y*${\;}_{i}$ is *m*${\;}_{i}$*.* For a pair of peaks *p*${\;}_{i}$ and *p*${\;}_{j}$ with i<j in the spectrum, if $m_{j}-m_{i}$ is the same as an amino acid or the modification of an amino acid in a protein, we can match *y*${\;}_{i}$ and *y*${\;}_{j}$ to the two nodes corresponding to the amino acid. Besides, following the same way as in Kou *et al.* (2017), we convert each mass value *m* in both PMG and SMG into an integer by using the formula ⌊m*274.335215⌋.

However, since the masses of peaks have errors, we cannot assume that $m_{j}-m_{i}$ is identical to the theoretical mass of an amino acid. Some kind of peak error handling is required. Usually, people use a value δ for error tolerance.

**Two ways to set error tolerance value** δ **:** There are two ways to set the error tolerance value δ (Kou *et al.* 2016, 2017, Zhan and Wang 2022). One way is to simply set δ=27 for every peak. The second method sets a value $\delta_{i}$ to be the error for *m*${\;}_{i}$ for each peak *y*${\;}_{i}$ and we call this kind of error tolerance peak-dependent error tolerance. We can calculate the peak dependent error tolerance for each peak *y*${\;}_{i}$ as follows.

1. For an original peak *y*${\;}_{i}$ with mass *m*${\;}_{i}$, $\delta_{i}=27+\frac{15}{1\;000\;000}m_{i}$.
2. For a complementary peak *y*${\;}_{i}$ with mass *m*${\;}_{i}$, $\delta_{i}=27+\frac{15}{1\;000\;000}(m_{i}\;+\;M)$, where *M* is the mass of the whole protein.
3. For any peak *y*${\;}_{i}$ with mass *m*${\;}_{i}$ larger than 5000, the corresponding $\delta_{i}$ should be further enlarged. An alternative way (Kou *et al.* 2016, 2017) to handle this kind of peaks is to add another two peaks with masses m−1.00235 and m+1.00235 in pre-process stage. After multiplying the factor of 274.335215, there are three peaks that are about 274.335215 away in the spectrum.

For item 3, there is a trade off between increasing the number of peaks in the spectrum and increasing the value of $\delta_{i}$. We observe that increasing the number of peaks is a better choice in terms of the speed of algorithms since the value of $\delta_{i}$ plays an important role for the algorithm with error correction. We denote $\delta_{\max}=\max_{i=0}^{n}\delta_{i}$.

The traditional method (Kou *et al.* 2017) computes an alignment between the two graphs (PMG and SMG). The alignment contains a list of nodes $x_{j_{1}},x_{j_{2}},\ldots ,x_{j_{k}}$ from the proteoform mass graph and a list of nodes $y_{i_{1}},y_{i_{2}},\ldots ,y_{i_{k}}$ from the spectrum mass graph such that for any two *consecutive* nodes $y_{i_{q}}$ and $y_{i_{q+1}}$ in the alignment, $m_{i_{q+1}}-m_{i_{q}}$ is in the range $\left[m_{q}\;-\;\delta ,m_{q}\;+\;\delta )\right]$, where *m*${\;}_{q}$ is the theoretical mass for a path between $x_{j_{q}}$ and $x_{j_{q+1}}$ in PMG. We refer to such kind of error tolerance method as *local edge tolerance methods*. The local edge tolerance methods may suffer from error accumulation if positive/negative errors occur for many edges in the alignment. For example, if $m_{i_{q+1}}-m_{i_{q}}=m_{q}+\delta$ for q=1,2,…k, then the mass between $y_{i_{1}}$ and $y_{i_{k}}$ is equal to the theoretical mass $\sum_{i=1}^{k}m_{i}$ plus kδ. Thus, in the alignment, there exists a pair of matched nodes, say, *y_1_* and *y*${\;}_{k}$ such that the mass between the two nodes has an error kδ compared to the theoretical mass in the PMG. Such a big mass error kδ will show that the obtained alignment is not reliable. The problem is so severe that it is necessary to use some kinds of heuristic methods to refine the obtained alignment (Kou *et al.* 2017).

### 2.1 Error correction alignment and the dynamic programming algorithm outline

To deal with the peak errors that may occur in the spectrum in a more accurate way, Zhan and Wang proposed a new model for error correction of peaks (Zhan and Wang 2022). They still use the proteoform mass graph (PMG) and the spectrum mass graph (SMG) to represent the database and the spectrum. They propose to have an error correction for each matched peak in the alignment so that after error correction the mass between any two matched peaks in SMG is identical to the corresponding theoretical mass in PMG.

Let *x_0_, x_1_*, …, *x*${\;}_{n}$ be the *n* nodes in a PMG *G* and *y_0_, y_1_*, …, *y*${\;}_{m}$ be the *m* nodes in a SMG *H*. An *error correction alignment* of *G* and *H* with size *r* is a sequence of *r* triples $\left(x_{j_{1}},y_{i_{1}},k_{i_{1}}),\;(x_{j_{2}},y_{i_{2}},k_{i_{2}}),\;\cdots ,\;(x_{j_{r}},y_{i_{r}},k_{i_{r}}\right)$ such that $m_{i_{q}}+k_{i_{q}}\;-\;(m_{i_{q-1}}\;+\;k_{i_{q-1}})=M_{j_{q-1},j_{q}}$, where $k_{i_{q}}\in (-\delta_{i_{q}},\delta_{i_{q}})$ is the error correction value for peak $y_{i_{q}},\;m_{i_{q}}$ is the mass for the peak $y_{i_{q}}$ and $M_{j_{q-1},j_{q}}$ is the mass of path between node $x_{j_{q-1}}$ and node $x_{j_{q}}$. Note that δ is the error tolerance value for the mass of the peak. The *error correction alignment problem* is to compute an error correction alignment between *G* and *H* with the maximum size.

Let T(i,j,k) be the maximum size of the alignments between the first *i* peaks in *H* and the first *j* nodes in *G* such that the corrected peak of *y*${\;}_{i}$ has the mass value $m_{i}+k$, and *y*${\;}_{i}$ in *H* matches *x*${\;}_{j}$ in *G*. Note that the initial value of T(i,j,k) is set to be 1 for all 0 ≤ i ≤ m, 0≤j≤n and $-\delta_{i}\;\leq \;k\;\leq \;\delta_{i}$.

When computing T(i,j,k) for every 0 ≤ i ≤ m, 0 ≤ j ≤ n and $-\delta_{i}\;\leq \;k\;\leq \;\delta_{i}$, a dynamic programming algorithm can be used to simplify the process. Let *d*(*s*, *j*) be the set of distinct masses for paths from *x*${\;}_{s}$ to *x*${\;}_{j}$ in *G*. For each mass m∈d(s,j), there is a list of nodes corresponding to peaks in *H* with mass values in the range $\left[(m_{i}\;+\;k)-m-\delta_{\max},(m_{i}\;+\;k)\;-\;m\;+\;\delta_{\max}\right]$. Let list(i,j,k,m) be such a sorted list. The peaks in this list can be matched to the node *x*${\;}_{s}$ in *G* under the condition that the corrected peak of *y*${\;}_{i}$ with mass $m_{i}+k$ matches the node *x*${\;}_{j}$ in *G*. Therefore, the following dynamic programming equation can be used for computing T(i,j,k):

(1) $$T(i,j,k)=\overset{j^{\prime }=0}{\underset{j^{\prime }=j-1}{\max}}\;\;\;\underset{m\in d(j^{\prime },j)}{\max}\underset{\left(i^{\prime },k^{\prime })\;\mathrm{satisfying}\;(2\right)}{\max}\{T(i^{\prime },j^{\prime },k^{\prime })+1\},$$

where condition (2) is as follows:

(2) $$\begin{array}{c} \;m-[(m_{i}+k)-m_{i^{\prime }}]=k^{\prime }. \end{array}$$

When computing list(i,j,k,m), let *C*(*i*, *j*) be the set of all list(i,j,k,m) and can be formulated as $C(i,j)=\{\mathrm{list}(i,j,k,m)|m\in \cup_{j^{\prime }=0}^{j^{\prime }=j-1}d(j^{\prime },j),k\in [-\delta_{\max},\delta_{\max}]\}$. Let $D=\cup_{j=0}^{j=n}\cup_{s=0}^{s=j-1}d(s,j)$ be the set of masses or subpaths in *G* that we consider for computation of alignments. Let $M=\{(m,i^{\prime },i)|i^{\prime }<i,y_{i},y_{i^{\prime }}\in H,m=m_{i}-m_{i^{\prime }}\}$ be the set of triples for masses differences *m* between any pair of nodes $\left(y_{i^{\prime }},y_{i}\right)$ in *H*. Then, *D* and *M* are both sorted in nondecreasing order. Going through the elements in sorted *D* and *M* once, we can create *C*(*i*, *j*) with all the lists list(i,j,k,m) sorted.

Moreover, for a specific peak *y*${\;}_{i}$, a node *x*${\;}_{j}$ and a path mass $m\in \cup_{j^{\prime }=0}^{j^{\prime }=j-1}d(j^{\prime },j)$ in *G*, one element in list(i,j,k,m) is enough to compute T(i,j,k) for all $k\in (-\delta_{i},\delta_{i})$ instead of using equation (1) for all $k\in (-\delta_{i},\delta_{i})$. [See Theorem 1 (Zhan and Wang 2022).] Thus, the total running time of computing all T(i,j,k) and finding the largest one is $O(nm\delta_{\max}+L)$ where *L* is the total size of $\cup_{j^{\prime }=0}^{j^{\prime }=j-1}d(j^{\prime },j)$.

The above algorithm is still too slow in practice. Thus, Zhan and Wang designed a local algorithm that can only compute an optimal alignment when the starting positions in both *G* and *H* are given. In this case, a sub-matrix of T(i,j,k) around the diagonal will be computed so that the running time and the memory usage are further reduced. The detailed algorithm can be found in the Supplementary Section S1A.

### 2.2 New fast algorithms

When computing T(i,j,k), for a peak *y*${\;}_{i}$, the corrected position $m_{i}+k$ of *y*${\;}_{i}$ is an integer in the range $\left[m_{i}-\delta_{i},m_{i}+\delta_{i}\right]$. When the next peak $y_{i+1}$ in the spectrum is close to *y*${\;}_{i}$, the two ranges $\left[m_{i}-\delta_{i},m_{i}+\delta_{i}\right]$ and $\left[m_{i+1}-\delta_{i+1},m_{i+1}+\delta_{i+1}\right]$ may have overlap. In this case, T(i,j,k) and $T(i+1,j,k^{\prime })$, where $m_{i}+k=m_{i+1}+k^{\prime }$, mean the same thing, i.e. a peak in the spectrum with mass $m_{i}+k=m_{i+1}+k^{\prime }$ matches *x*${\;}_{j}$ in *G*. It does not matter whether *y*${\;}_{i}$ or $y_{i+1}$ is corrected to the position $m_{i}+k=m_{i+1}+k^{\prime }$ since Theorem 1 in Zhan and Wang (2022) ensures that $T(i,j,k_{i})$ is always equal to $T(i+1,j,k_{i+1})$ under the condition $m_{\mathrm{small}}>4\delta_{\max}$, where *m*${\;}_{\mathrm{small}}$ is the smallest mass of all the (modified or unmodified) residues. This condition is true in practice for different error tolerance settings (Zhan and Wang 2022).

**Figure 1.**
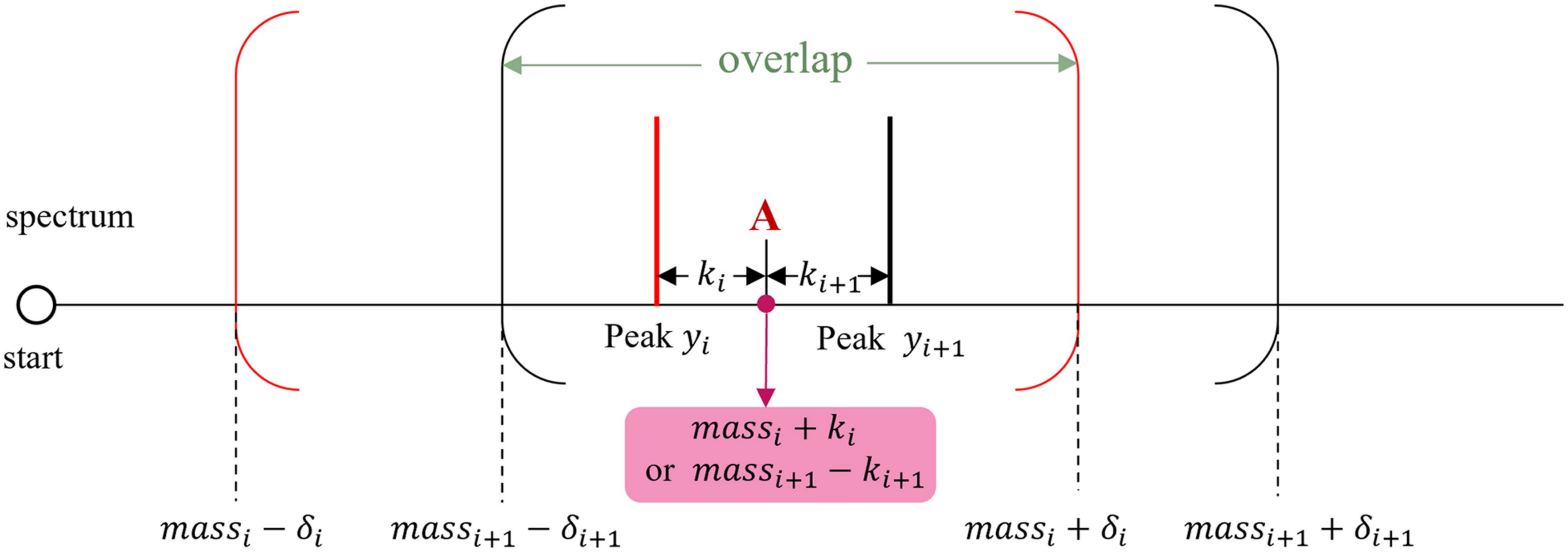
The figure illustration example for the error tolerance overlaps. The range between the position $\mathrm{mas}s_{i+1}-\delta_{i+1}$ and $\mathrm{mas}s_{i}+\delta_{i}$ is the overlap and the positions like the point *A* in it will be computed twice.

Thus, each integer in the overlapped range $\left[m_{i}-\delta_{i},m_{i}+\delta_{i}]\cap [m_{i+1}-\delta_{i+1},m_{i+1}+\delta_{i+1}\right]$ is used twice in the computation process of T(i,j,k)s. See Fig. 1. Therefore, we can make the algorithm faster if every integer in the overlapped range is used once during the whole process of computing T(i,j,k)s.

To reduce such redundant computing, we propose an algorithm to delete overlaps when computing T(i,j,k) and ensure every available position for peaks is computed only once. In the new algorithm, for each peak, we define two variables to indicate the lower and upper bounds of the range, where the correct peak position should be. Let $\delta_{i}^{-}$ denote the largest negative error tolerance for peak *y*${\;}_{i}$*.* Similarly, let $\delta_{i}^{+}$ denote the largest positive error tolerance for peak *y*${\;}_{i}$*.* The initial values of $\delta_{i}^{-}$ and $\delta_{i}^{+}$ for peak *y*${\;}_{i}$ are both $\delta_{i}$ before deleting overlaps.

For any consecutive peaks *y*${\;}_{i}$ and *y*${\;}_{j}$, if the range $\left[m_{i}-\delta_{i}^{-},m_{i}+\delta_{i}^{+}\right]$ is completely included in the range $\left[m_{j}-\delta_{j}^{-},m_{j}+\delta_{j}^{+}\right]$, then *y*${\;}_{i}$ can be deleted from the spectrum at the beginning of this algorithm.

We then re-calculate the ranges for peak *y*${\;}_{i}$ and $y_{i+1}$ according to the principles illustrated in the Supplementary Section S1B.

Now, when we compute T(i,j,k), the range of the element *k* is reduced and the total size of T(i,j,k)s we need to compute is also decreased. We still use Equation (1) to compute T(i,j,k). Thus, the total running time is O(nmq+L), where *q* is the largest size of $\delta_{i}^{-}+\delta_{i}^{+}$ for all peaks.

Besides, in the process creating *C*(*i*, *j*), we also change the formulation of *C*(*i*, *j*) by the updated error tolerance for every peak as $C(i,j)=\{\mathrm{list}(i,j,k,m)|m\in \cup_{j^{\prime }=0}^{j^{\prime }=j-1}d(j^{\prime },j),k\in [-\delta_{i}^{-},\delta_{i}^{+}]\}$. This updated formulation can also decrease the size of *C*(*i*, *j*) and further reduce the running time for creating *C*(*i*, *j*).

## 3 Results

### 3.1 Datasets

Here, we use a dataset generated from Escherichia coli (EC) K-12 MG1655 cells. The protein database was downloaded from UniProt (Proteome ID: UP000000625) and included 4438 protein entries associated with this proteome with *F* plasmid removed. For MS and MS/MS spectra, we use the raw features downloaded from Kou *et al.* (2017) and further process the data following the methods described in Kou *et al.* (2017). After processing, we obtained 4054 top-down MS/MS spectra, where 2027 are collision-induced dissociation (CID) MS/MS spectra and the other 2027 are electron-transfer dissociation (ETD) MS/MS spectra. For this EC dataset (database), three mutations were used as variable PTMs and the three modifications are: lysine (K) to cysteine (C) (UNIMOD Accession number: 1132), threonine (T) to alanine (A) (UNIMOD Accession number: 659) and valine (V) to glycine (G) (UNIMOD Accession number: 672). A txt format file was generated based on these three pre-defined mutations as part of the input. Users can provide their own predefined mutations to replace the txt format file. The original EC database was modified based on the txt format file to form the final proteoform database for database search. For the EC protein database, the protein size (the total number of amino acids) ranges from 31 to 2001, while the spectrum size (the total number of peaks) ranges from 22 to 4604 among 4054 top-down MS/MS spectra.

### 3.2 Speed and memory of (local) diagonal alignment version

The best-known method with error correction is TopMGRefine (Zhan and Wang 2022), where they can only provide a diagonal alignment version reporting an optimal local alignment due to the high time/space complexity of their algorithm. The diagonal alignment version requires users to input the starting positions of the alignment and has a constraint that the two masses from the two starting positions in the alignment to any pair of aligned nodes are roughly the same. To illustrate the time/space complexity of the new algorithm, we start with the diagonal alignment version and follow the experiment processes in Zhan and Wang (2022).

Similar to TopMGRefine, since diagonal alignment needs the starting positions as part of the input, we use TopMG (Kou *et al.* 2017) to align all the 4054 spectra with all the proteins for the whole EC dataset. Note that, TopMG first uses a filtering method to obtain a few candidate proteins with high scores for each spectrum and then uses an alignment algorithm to further align the spectrum with each of the selected candidates and report the protein and the corresponding alignment with the best score. We refer to this version as *TopMG with filtering*.

Among 4054 spectra, TopMG reported 2817 spectra that can be successfully aligned to some proteins in the database. Then we choose the protein with the largest alignment size generated by TopMG for each of the 2817 spectra to form protein and spectrum pairs for further refined alignments using TopMGRefine and TopMGFast.

The experiments were performed on a cluster with 200 GB of memory. For the 2817 protein and spectrum pairs, both TopMGRefine and TopMGFast obtain identical local alignment results. The total running time of the 2817 protein and spectrum pairs for TopMGrefine and TopMGFast is 4760 and 1715 min, respectively.

The largest input instance is the protein sp|P76347|YEEJ_ECOLI with 2001 residues and the spectrum (ID: 3260) with 4604 peaks. For this largest input instance, the starting position for protein and the starting position for the spectrum given by TopMG are 813 and 0, respectively. In this case, the space required by TopMGRefine and TopMGFast is 102G and 80G, respectively. The alignment size obtained by both TopMGRefine and TopMGFast is 83. The running time for TopMGFast and TopMGRefine is 21.4 and 56.9 min, respectively. Again, the running time of TopMGFast is about 1/3 of that for TopMGRefine. Therefore, we can see that TopMGFast and TopMGRefine can obtain the same results, where TopMGFast needs much less running time and memory space.

### 3.3 Preliminary performance of TopMGFast for global alignment

Since TopMGFast needs less memory space, it is possible to do global alignment even for the largest input instance, where TopMGRefine cannot do that. For global alignment, we do not need to give the starting positions of the alignment for a pair of protein and spectrum. Therefore, we can try to compute the final accurate alignment result without using the roughly estimated starting positions of the alignment.

In the rest of this subsection, we will compare the alignment algorithms for TopMG and TopMGFast using 4054 top-down MS/MS spectra and 4438 proteins in the whole EC database. Since both the protein and spectrum databases are huge compared to the slow speeds of the alignment algorithms for both TopMG and TopMGFast, we first run the filtering algorithm in TopMG to obtain a few candidates for each spectrum, and for the same set of obtained candidate proteins, run the alignment algorithms for both TopMG and TopMGFast with the same spectrum to get the best alignment results.

Among 4054 spectra in the whole EC dataset, TopMG reported 2817 spectra with at least one corresponding protein candidate and the total number of reported corresponding protein candidates is 31 481. Each reported spectrum corresponds to a few candidate proteins with large alignment scores. We then align them with each other and report the final alignments by using TopMGFast directly. This time, no starting points are required. Besides, we used two kinds of error tolerances for peaks when computing alignment results for both TopMG and TopMGFast, one is constantly equal to 27 and the other is the peak-dependent error tolerance computed in Section Methods. The experiments were also performed on a cluster with 200 GB of memory.

#### 3.3.1 Results for error tolerance 27

The results with error tolerances 27 are shown in Tables 1 and 2. As shown in Table 1, the running time of TopMGFast is 8702 min which is about 6.6 times that of TopMG when setting the alignment error tolerance to be 27. Among 2817 selected spectra, TopMG and TopMGFast can report the same proteins for 1396 spectra and different proteins for the other 1421 spectra, respectively. Furthermore, among 1396 spectra reporting the same proteins from TopMG and TopMGFast, 1082 spectra report roughly the same alignment locations (resulting in alignments with overlaps), while 314 spectra report completely different alignment locations.

**Table 1.** The comparisons between TopMG and TopMGFast using error tolerances equals 27 without diagonal optimization on the filtered EC dataset.

Algorithm	Running time (min)	Alignment size	Same protein^b^	Different proteins^a^
TopMG	1320	Total no. of matches	23.19	13.34
		No. of 1 residue matches^c^	11.04	2.75
		No. of 2 residues matches	2.97	1.31
		No. of ≥3 residues matches	9.18	9.28
TopMGFast	8702	Total no. of matches	24.61	17.57
		No. of 1 residue matches	11.49	3.08
		No. of 2 residues matches	3.33	1.78
		No. of ≥3 residues matches	9.79	12.71

a For the reported proteins from TopMG and TopMGFast among 2817 spectra, the same reported protein can be obtained for 1396 spectra and different reported proteins can be obtained for 1421 spectra, respectively.

b Among 1396 spectra reporting the same proteins from TopMG and TopMGFast, 1082 spectra report overlap alignment results while 314 spectra report completely different alignment results.

c Each pair of adjacent peaks in the alignment corresponds to a sub-path in *G*. ‘*i* residue matches’ means that such a sub-path contains *i* residues for *i *=* *1, 2, 3.

**Table 2.** MME and AME comparisons between TopMG and TopMGFast for 2817 alignments when the predefined error tolerance δ=27.

Algorithm	Mass error	Total	The same protein	Different proteins
TopMG	Average MMEs	49.43	36.06	66.16
	Average AMEs	17.25	11.56	24.37
TopMGFast	Average MMEs	35.14	26.87	45.49
	Average AMEs	11.42	7.77	15.99

For both 1396 (reporting identical proteins) and 1421 (reporting different proteins) spectra groups, the numbers of matches obtained from TopMGFast are slightly larger than that of TopMG. (24.61 versus 23.19 and 17.75 versus 13.34, respectively.) Consequently, the average numbers of one residue and two residue matches for reported alignments obtained from TopMGFast are also slightly larger than those obtained from TopMG. Since mass matches of one or two residues are more reliable than mass matches corresponding to a sum of a large number of residues, this might be evidence that the alignments reported by TopMGFast are more reliable than those of TopMG. It is interesting to observe that the number of 1 residue matches is slightly <50% of total matches.

The reason that TopMGFast can obtain more matches than TopMG is that TopMGFast requires that the difference between the locations of a peak and the corrected peak is bounded by 27, where TopMG requires that the mass between two consecutive matched peaks is at most 27 away from the corresponding mass in the protein. Thus, it is possible that the tolerated error of the mass between two consecutive matched peaks for TopMGFast is larger than that of TopMG. However, TopMGFast can ensure that there is no error for a mass between any pair of matched peaks after error correction.

To further compare the quality of resulting alignments, we use two measures, the maximum mass error (MME) between two matched peaks in an alignment and the average mass error (AME) between all pairs of matched peaks. The maximum mass error (MME) between two matched peaks in an alignment is the largest error among all the $\left(\begin{array}{c} n\\ 2 \end{array}\right)$ pairs of *n* matched peaks in an alignment. The average mass error (AME) between two matched peaks in an alignment is the average error (comparing to the theoretical masses in the protein database) among all the $\left(\begin{array}{c} n\\ 2 \end{array}\right)$ pairs of *n* matched peaks in an alignment.

**Figure 2.**
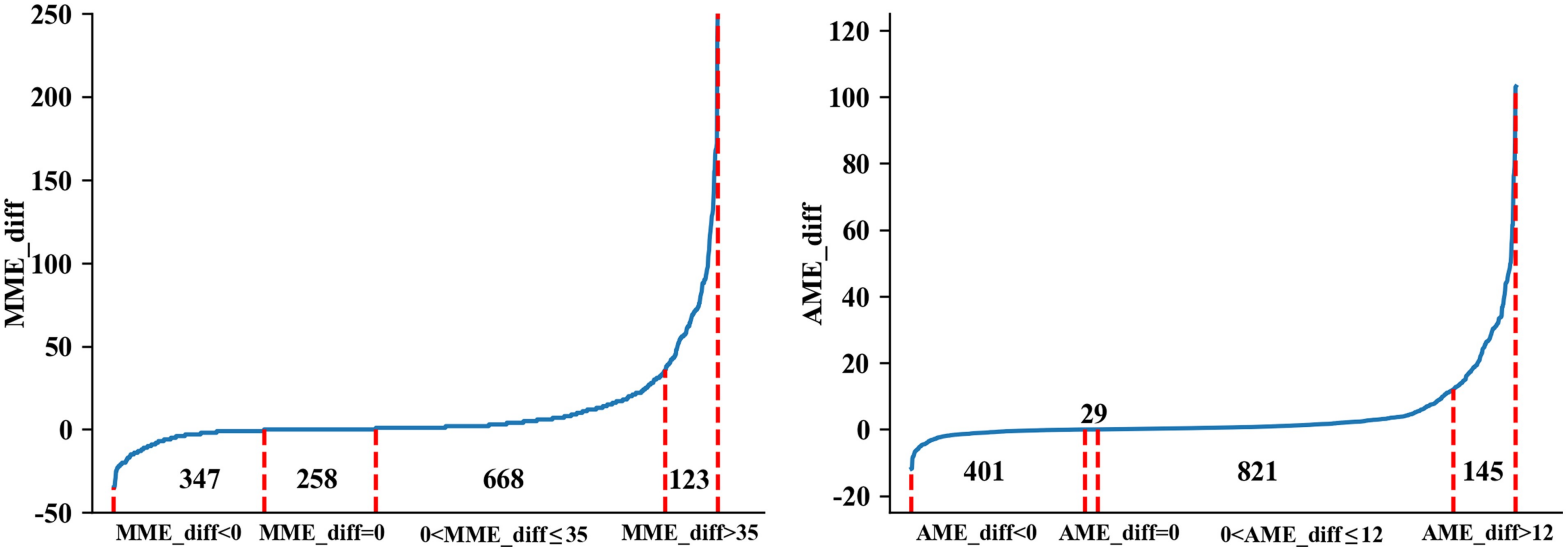
The differences between TopMG’s and TopMGFast’s MME and AME values for those spectra reporting the same proteins when the predefined error tolerance δ=27. Here, MME/AME_diff=TopMG′s MME/AME−TopMGFast′s MME/AME. The 1396 spectra have been sorted by MME/AME_diff in nondecreasing order.

Here, we plot the differences between TopMG’s and TopMGFast’s MME and AME values (MME/AME_diff) for those spectra, where both methods report the same proteins in Fig. 2. MME/AME_diff can be computed by the formula MME/AME_diff=TopMG′s MME/AME-TopMGFast′s MME/AME. As shown in Fig. 2, 1396 spectra that report the same proteins have been sorted by MME/AME_diff in nondecreasing orders. For these 1396 spectra, there are 347 spectra in which −35≤MME_diff<0 in the left figure. This means that TopMGFast’s alignment results have worse MME values compared with TopMG’s alignment results for these 347 spectra. The worst case is the spectrum that MME_diff=−35. Similarly, the numbers of spectra for the conditions MME_diff=0, 0<MME_diff≤35 and MME_diff>35 are 258, 668, and 123, respectively. Hence, there are 791 spectra in which TopMGFast’s alignment results are better than TopMG’s alignment results. The best case is the spectrum that MME_diff=252. The MME_diff is unreasonably large since TopMG has no constraint for any two matched peaks. This makes the TopMG’s alignment result unreliable. For AME_diff, it also exhibits the same pattern.

Besides, for all 2817 spectra, the average MME and AME values are given in Table 2. The detailed diagrams are illustrated in the Supplementary Section S2A. As shown in Supplementary Fig. S5, there is an upper bound 2δ=2×27=54 for the MMEs reported by TopMGFast for all the 2817 alignments, while we can clearly observe that TopMG reported many alignments with unreasonably large MMEs. Note that, one pair of peaks with an unreasonably large error indicates that the alignment is not reliable.

#### 3.3.2 Results for peak-dependent error tolerance

**Figure 3.**
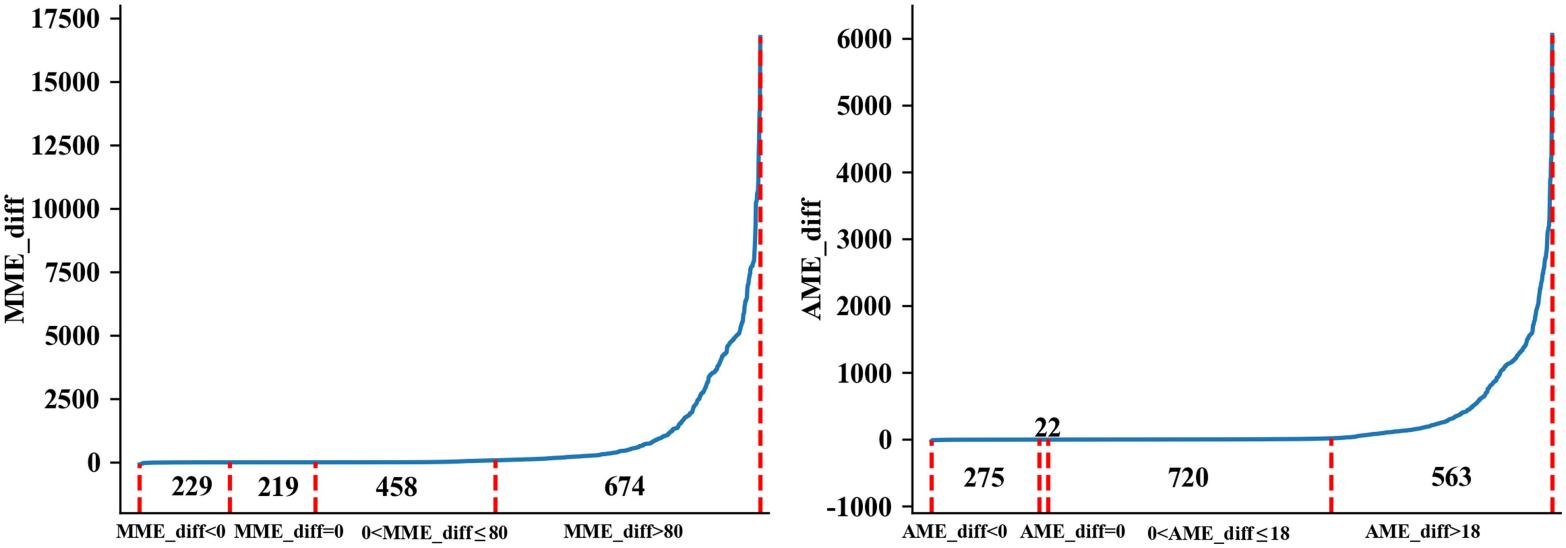
The differences between TopMG’s and TopMGFast’s MME and AME values for those spectra reporting the same proteins when using the peak-dependent error tolerance. Here, MME/AME_diff=TopMG′s MME/AME−TopMGFast′s MME/AME. The 1580 spectra have been sorted by MME/AME_diff in nondecreasing order.

Tables 3 and 4 illustrate the results for the complex error tolerances. As we can see, the running time of TopMGFast without diagonal optimization is 34 240 min which is about 3.3 times that of TopMG. Among 2817 selected spectra, TopMG and TopMGFast can report the same proteins for 1580 spectra and different proteins for 1237 spectra, respectively. Furthermore, among 1580 spectra reporting the same proteins from TopMG and TopMGFast, 1150 spectra report roughly the same locations while 430 spectra report completely different locations. However, the alignment size obtained by TopMGFast is slightly smaller than that obtained from TopMG. The reason is that TopMGFast requires that the mass between any matched peaks (after peak error correction) be identical to the corresponding theoretical mass in the protein database, while TopMG requires any two consecutive matched peaks to have a mass error at most δ. TopMG has a high chance to generate alignments with huge MMEs. Figure 3 illustrates MME/AME_diff for those spectra, where both methods report the same proteins when we use the peak-dependent error tolerance. The worst cases are MME=−80 and AME=−17.8. The best cases are MME* *=* *16 755 and AME* *=* *6058.9. The huge MME/AME_diff values indicate the unreliability of TopMG, and our algorithm TopMGFast can generate more accurate alignments.

**Table 3.** The comparisons between TopMG and TopMGFast using the same peak-dependent error tolerances without diagonal optimization on the filtered EC dataset.

Algorithm	Running time (min)	Alignment size	Reporting the same protein^b^	Reporting different proteins^a^
TopMG	10 371	Total no. of matches	35.09	34.20
		No. of 1 residue matches	16.96	10.71
		No. of 2 residues matches	5.11	4.97
		No. of ≥3 residues matches	13.02	18.52
TopMGFast	34 240	Total no. of matches	32.64	29.78
		No. of 1 residue matches	15.02	6.46
		No. of 2 residues matches	4.73	3.95
		No. of ≥3 residues matches	12.89	19.37

a For the reported proteins from TopMG and TopMGFast among 2817 spectra, the same reported protein can be obtained for 1580 spectra and different reported proteins can be obtained for 1237 spectra, respectively.

b Among 1580 spectra reporting the same proteins from TopMG and TopMGFast, 1150 spectra report overlaps alignment results while 430 spectra report completely different alignment results.

**Table 4.** The error comparisons between TopMG and TopMGFast for 2817 alignments when using the peak-dependent error tolerance with overlaps deleted.

Algorithm	Mass error	Total	The same protein	Different proteins
TopMG	Average MMEs	1043.95	823.05	1351.19
	Average AMEs	326.73	250.55	432.68
TopMGFast	Average MMEs	159.35	115.36	220.53
	Average AMEs	43.20	28.84	63.18

Besides, for all 2817 spectra, the average MME and AME values are given in Table 4. The detailed diagrams are illustrated in Supplementary Section S2B.

#### 3.3.3 Comparison of reported alignment locations with the two error tolerance methods

We also compared the reported proteins with the two different error tolerance methods. The results are shown in Table 5. As we can see, both TopMG and TopMGFast reported different proteins for large amounts of spectra when using two kinds of error tolerances. Besides, according to Tables 1 and 3, for the averaged total number of reported matches obtained from all spectrum and proteoform candidate pairs, both TopMG and TopMGFast can report more matches when using peak-dependent error tolerance. This may be evidence that using the peak-dependent error tolerance rather than the immutable value 27 is a better choice for handling the peak errors.

**Table 5.** The comparisons of reported proteins between two kinds of error tolerances for TopMG and TopMGFast.

Algorithm	Error tolerance	No. of matches	RT(min)	Different proteins	The same protein
TopMG	27	18.22	1320	1343	1474
	Complex	34.70	10 371		
TopMGFast	27	21.06	8702	966	1851
	Complex	31.38	34 240		

### 3.4 Prediction accuracy evaluation

The preliminary performance analysis in the above subsection shows that TopMG and TopMGFast report different proteins in many cases for the same spectrum even if they use the same set of filtered candidate proteins. To illustrate the quality of the alignments for the two different alignment algorithms, we delete the filter step and directly use the two alignment algorithms to search the protein database. Without the filtering algorithm, both TopMG and TopMGFast are slow. Thus, we reduce the size of the database as follows. We randomly select 700 proteins from the same protein database mentioned above to form the testing protein database. Then we randomly select 100 proteins from the testing protein database and generate a simulated spectrum with 5 modifications for each of them using MaSS-Simulator (Awan and Saeed 2018). In this way, for each of the simulated spectra, we know the real corresponding (modified) protein.

For the MaSS-Simulator, a protein sequence file and a modification file should be provided as the input files. Here, we use the sample modification file given by the paper (Awan and Saeed 2018) with four kinds of mutations: Oxidation (UNIMOD Accession number: 35), Deamidation (UNIMOD Accession number: 7), Phosphorylation (UNIMOD Accession number: 21), and Carbamidomethylation (UNIMOD Accession number: 4). Besides, each protein is set to have five modifications in the generated spectrum.

Now, we have 100 simulated spectra with their known real corresponding proteins and a protein database with 700 proteins. The original database has >4000 proteins. The reason that we do not use the whole database is that the speeds of both TopMG and our method are very slow to directly search the whole database.

We use both TopMG and our method to directly align the 100 simulated spectra against the 700 proteins in the database and report the proteins with the best alignment score as the search results. According to the alignment results, TopMG reported 94 real corresponding proteins for the 100 cases. Thus, the prediction accuracy for TopMG is 94%. Our method, TopMGFast, reported 100 real corresponding proteins for the 100 cases and the prediction accuracy is 100%.

**Case study:** TopMG failed the case, where the real corresponding protein is sp | P0A235 | RFC_SALTY. For this case, TopMG reported protein sp | P26465 | FLII_SALTY with 53 matched peaks in the alignment. We checked the errors between every two adjacent matched peaks in this alignment for both TopMG and TopMGFast. We found that there are many consecutive negative errors (the first few errors are: [4, −1, −20, −9, −16, −24, …]) in the alignment reported by TopMG. These consecutive negative errors make the maximum mass error for TopMG become 121 which is much larger than the user-defined error tolerance 27. However, the error between any two matched peaks in the alignment reported by TopMGFast is strictly no more than 2×27=54. Maybe this is the reason why TopMG reported the wrong protein and the evidence that error correction methods such as TopMGFast are more reliable than TopMG.

To test the performance accuracy for local alignment, we randomly select a peptide from each of the above 100 selected proteins. The lengths of those selected peptides are from 23 to 154. Each peptide contains three modifications. We then generate 100 simulated spectra for the 100 peptides and use both TopMG and TopMGFast to search the database containing 700 proteins. TopMG reported 98 real corresponding proteins with correct locations for the 100 cases. The prediction accuracy for TopMG is 98%. TopMGFast reported 100 correct spectra and the prediction accuracy is 100%.

## 4 Conclusion

We propose a faster algorithm for the error correction alignment of spectrum mass graph and proteoform mass graph problem and produce a program package TopMGFast in C++. The newly designed algorithms require less space and running time so that we are able to compute global optimal alignments for the two input mass graphs in a reasonable time. According to the experiment results for both local alignments and global alignments, our new algorithm can obtain more reliable alignments within a reasonable time. Besides, this is the first time that global optimal error correction alignments can be obtained using real datasets. Still, it is an interesting open problem to design better error handling methods.

## Supplementary Material

btae149_Supplementary_Data

## Data Availability

The data underlying this article are available at https://github.com/Zeirdo/TopMGFast.
