# Supplementary material for: Fast peak error correction algorithms for proteoform identification using top-down tandem mass spectra
Source: Bioinformatics. 2024 Mar 18;40(4):btae149. doi: 10.1093/bioinformatics/btae149 (PMC11212493; doi:10.1093/bioinformatics/btae149)
Supplement: btae149_Supplementary_Data [file btae149_supplementary_data.pdf]

Supplementary Material

1. ALGORITHM

A. Diagonal alignment with error corrections

Let x_{j_0} be the starting position in the proteoform mass graph G and y_{i_0} the starting position in the spectrum mass graph H. Besides, let $M(j_0, j)$ be the total mass of the unique path from x_{j_0} to x_j containing all the black edges in G. Let α^+ be the largest positive mass difference between a colored edge and the black edge connecting a consecutive pair of nodes from x_{j_0} to x_j in G. Similarly, let α^- be the smallest negative mass difference between a colored edge and the black edge connecting a consecutive pair of nodes from x_{j_0} to x_j. Therefore, the main idea of this diagonal alignment is that only peaks with mass $mass_i$ such that $mass_i - mass_{i_0}$ is in the range $[M(j_0, j) + r\alpha^-, M(j_0, j) + r\alpha^+]$ will be considered as valid peaks for a specific node x_{j_r} and can be further used for computing $T(i, j, k)$.

B. Principles for re-calculating the peak error ranges

When re-calculating the ranges for peak y_i and y_{i+1}, we should follow the three cases illustrated in Figure.S2 to Figure.S5.

Case 1: The mass m_{i+1} of peak y_{i+1} is in the range $[m_i - \delta_i^-, m_i + \delta_i^+]$, and $m_i - \delta_i^- < m_{i+1} - \delta_{i+1}^- \leq m_i + \delta_i^+ < m_{i+1} + \delta_{i+1}^+$. Thus, $m_{i+1} \leq m_i + \delta_i$. (See Fig. S1.)

To remove the overlap, we set the new values as follows:

$$\delta_i^+ = (m_{i+1} - 1) - m_i, \tag{S1}$$

$$\delta_{i+1}^- = 0. \tag{S2}$$

The values of δ_i^- and δ_{i+1}^+ remain the same. Therefore, the new range for peak y_i is $[m_i - \delta_i^-, m_{i+1} - 1]$, while the new range for peak y_{i+1} is $[m_{i+1}, m_{i+1} + \delta_{i+1}^+]$.

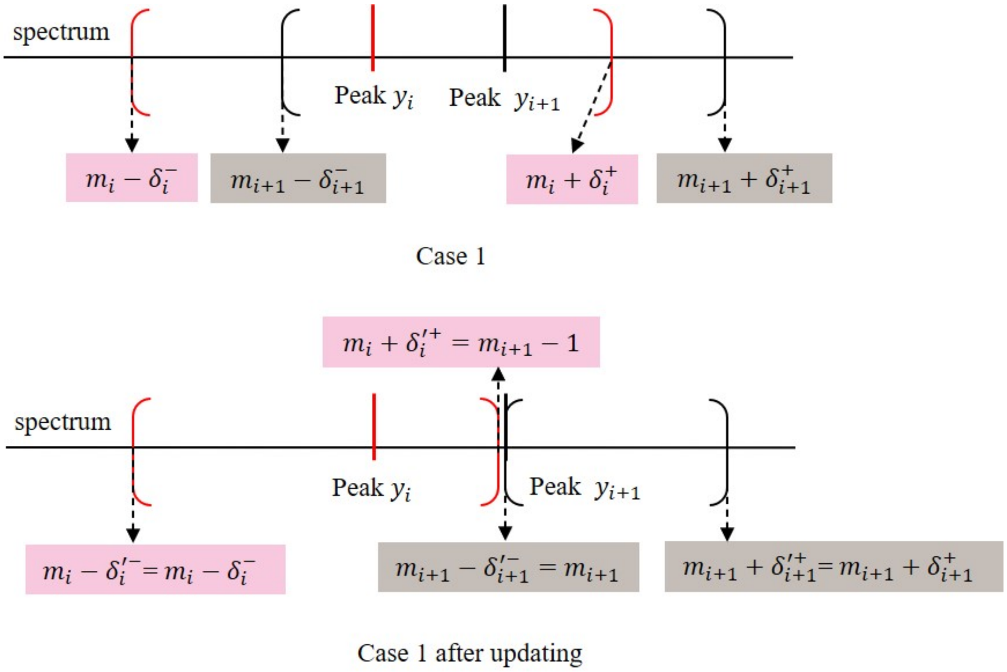

Fig. S1. The figure illustrations for **Case 1** and **Case 1 after updating**. The range for peak y_i is included in the red brackets, while the range for peak y_{i+1} is included in the black brackets.

Case 2: The mass m_{i+1} of peak y_{i+1} is in the range $[m_i - \delta_i^-, m_i + \delta_i^+]$, and $m_{i+1} - \delta_{i+1}^- < m_i - \delta_i^- < m_{i+1} + \delta_{i+1}^+ < m_i + \delta_i^+$.

For this case, there are two sub-cases to consider.

Case 2.1: The ranges for peak y_{i-1} and peak y_{i+1} have no overlap. Thus, $m_{i-1} + \delta_{i-1}^{'+} < m_{i+1} - \delta_{i+1}^-$. (See Fig. S2.)

To remove the overlap included in the ranges of peak y_i and peak y_{i+1}, we set the new values as follows:

$$\delta_i^{'-} = m_i - (m_{i+1} - \delta_{i+1}^-), \tag{S3}$$

$$\delta_i^{'+} = (m_{i+1} - 1) - m_i, \tag{S4}$$

$$\delta_{i+1}^{'-} = 0, \tag{S5}$$

$$\delta_{i+1}^{'+} = (m_i + \delta_i^+) - m_{i+1}. \tag{S6}$$

Therefore, the new range for peak y_i is $[m_{i+1} - \delta_{i+1}^-, m_{i+1} - 1]$, while the new range for peak y_{i+1} is $[m_{i+1}, m_i + \delta_i^+]$. (See Fig. S2 after updating.)

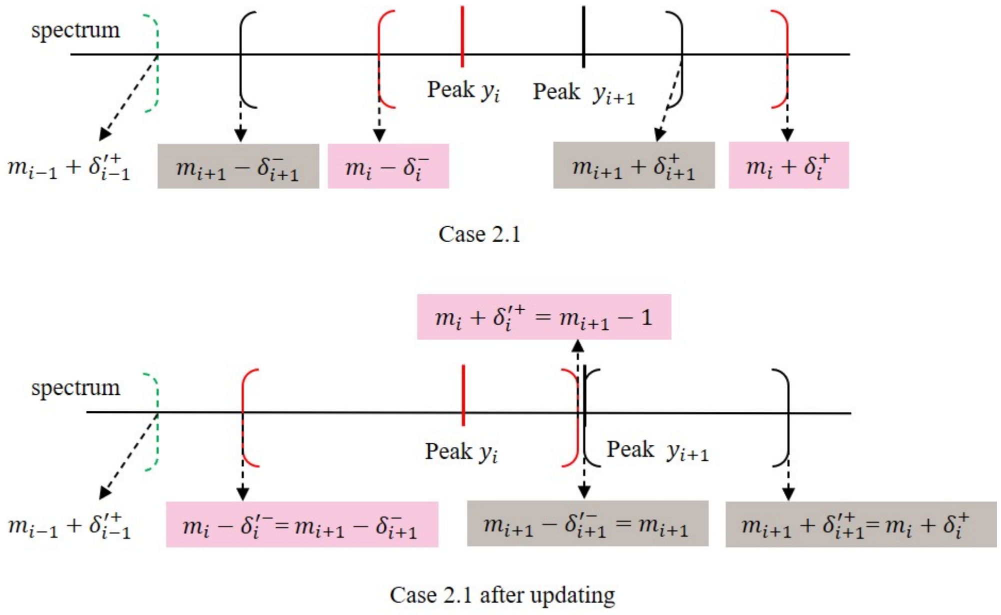

Fig. S2. The figure illustrations for **Case 2.1** and **Case 2.1 after updating**. The range for peak y_i is included in the red brackets, while the range for peak y_{i+1} is included in the black brackets. The green dotted bracket represents the updated right boundary of the range for the peak y_{i-1}.

Case 2.2: The ranges for peak y_{i-1} and peak y_{i+1} have an overlap. Thus, we have $m_{i+1} - \delta_{i+1}^- <= m_{i-1} + \delta_{i-1}^{'+}$. Moreover, $(m_i - \delta_i^-) > (m_{i-1} + \delta_{i-1}^{'+})$ assuming we have done the updating process for peaks y_{i-1} and y_i. (See Fig. S3.)

If $(m_i - \delta_i^-) - (m_{i-1} + \delta_{i-1}^{'+}) = 1$, the range $[m_{i+1} - \delta_{i+1}^-, m_{i+1} + \delta_{i+1}^+]$ of peak y_{i+1} is completely included in the range $[m_{i-1} - \delta_{i-1}^-, m_{i-1} + \delta_{i-1}^+] \cup [m_i - \delta_i^-, m_i + \delta_i^+]$. Thus peak y_{i+1} can be deleted from the spectrum. Therefore, we only have to consider $(m_i - \delta_i^-) - (m_{i-1} + \delta_{i-1}^{'+}) > 1$.

To remove overlaps, the values of $\delta_i^{'+}$, $\delta_{i+1}^{'-}$, $\delta_{i+1}^{'+}$ are updated in the way as in Case 2.1, and the value of δ_i^{i-} need to be updated as follows:

$$\delta_i^{i-} = m_i - (m_{i-1} + \delta_{i-1}^{'+} + 1). \tag{S7}$$

Therefore, the new range for peak y_i is $[m_{i-1} + \delta_{i-1}^{'+} + 1, m_{i+1} - 1]$, while the new range for peak y_{i+1} is $[m_{i+1}, m_i + \delta_i^+]$. (See Fig. S3 after updating.)

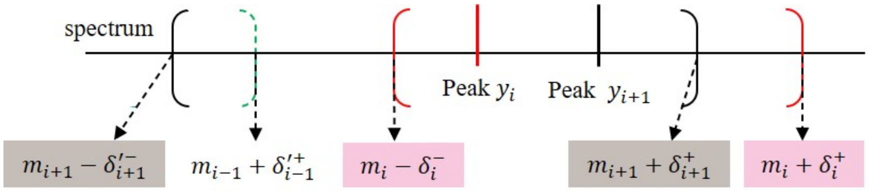

Case 2.2

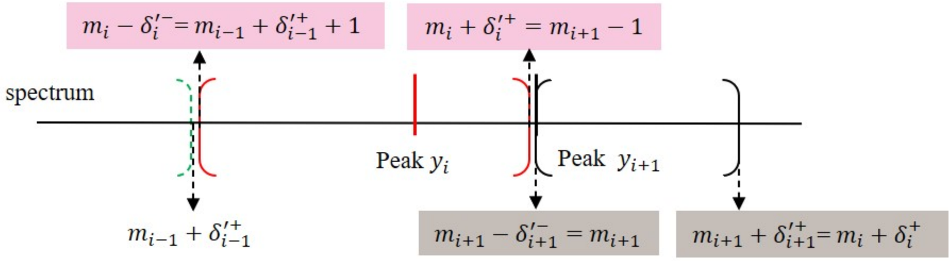

Case 2.2 after updating

Fig. S3. The figure illustrations for **Case 2.2** and **Case 2.2 after updating**. The range for peak y_i is included in the red brackets, while the range for peak y_{i+1} is included in the black brackets. The green dotted bracket represents the updated right boundary of the range for the peak y_{i-1}.

Case 3: The mass m_{i+1} of peak y_{i+1} is not in the range $[m_i - \delta^-_i, m_i + \delta^+_i]$, but $m_i + \delta^+_i > m_{i+1} - \delta^-_{i+1}$. Thus, we have $m_{i+1} > m_i + \delta_i$. Moreover, we can assume that $m_{i+1} - \delta^-_{i+1} > m_i - \delta^-_i$. Since the range for peak y_i will not be completely included in the range for peak y_{i+1}. Thus, $m_i - \delta^-_i < m_{i+1} - \delta_{i+1} - \delta^-_{i+1} < m_i + \delta^+_i < m_{i+1} + \delta^+_{i+1}$. (See Figure S4.)

To remove the overlap, we set the new value of δ^-_{i+1} as follows:

$$\delta'^{-}_{i+1} = m_{i+1} - (m_i + \delta^+_i + 1). \tag{S8}$$

The values of δ^-_i, δ^+_i and δ^+_{i+1} remain the same. Therefore, the new range for peak y_i is $[m_i - \delta^-_i, m_i + \delta^+_i]$, while the new range for peak y_{i+1} is $[m_i + \delta^+_i + 1, m_{i+1} + \delta^+_{i+1}]$. (See Figure S4 after updating.)

Now, when we compute $T(i,j,k)$, the range of the element k is reduced and the total size of $T(i,j,k)$s we need to compute is also decreased. We still use equation (**??**) to compute $T(i,j,k)$. Thus, the total running time is $O(nmq + L)$, where q is the largest size of $\delta^-_i + \delta^+_i$ for all peaks.

Besides, in the process creating $C(i,j)$, we also change the formulation of $C(i,j)$ by the updated error tolerance for every peak as $C(i,j) = \{list(i,j,k,m) | m \in \cup_{j'=j-1}^{j'=0} d(j',j), k \in [-\delta^-_i, \delta^+_i]\}$. This updated formulation can also decrease the size of $C(i,j)$ and further reduce the running time for creating $C(i,j)$.

2. RESULTS

A. The MME and AME values for all 2817 spectra when the predefined error tolerance $\delta = 27$

The MMEs and AMEs for all the best 2817 alignments reported by TopMG and TopMGFast can be seen in an intuitive way in Fig. S5. All 2817 alignments have been sorted by MMEs and AMEs of TopMGFast in non-decreasing orders. Besides, for those alignments with the same MMAs and AMEs reported by TopMGFast, we further sorted them by the MMEs and AMEs of TopMG in non-decreasing orders. As shown in Fig. S5, there is an upper bounder 2δ (here is $2 * 27 = 54$) for the MMEs reported by TopMGFast for all the 2817 alignments. This upper bounder comes from the constraint of TopMGFast that the mass between any two matched peaks (after peak error correction) is identical to the theoretical mass in the protein database. There is no such kind of constraint for TopMG and we can clearly observe that there exist so many alignments that the MMEs reported by TopMG are unreasonably large. Note that, one pair of peaks with

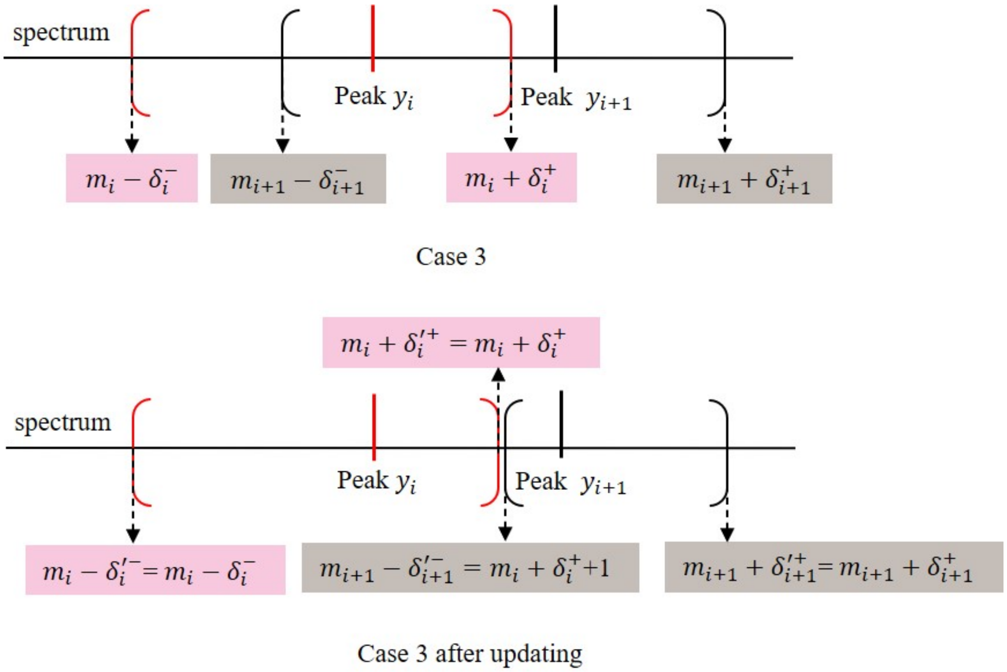

Fig. S4. The figure illustrations for **Case 3** and **Case 3 after updating**. The range for peak y_i is included in the red brackets, while the range for peak y_{i+1} is included in the black brackets.

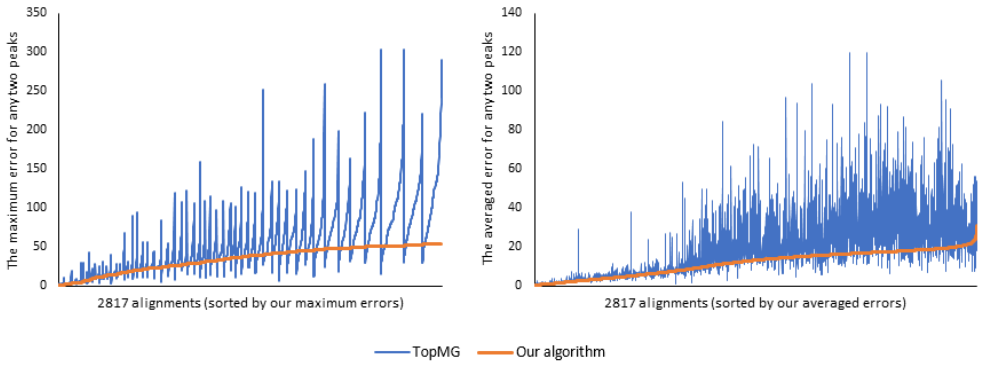

Fig. S5. MME and AME comparison curves between TopMG and TopMGFast for 2817 alignments when the predefined error tolerance $\delta = 27$. The 2817 alignment has been sorted by MMEs and AMEs of TopMGFast in non-decreasing order.

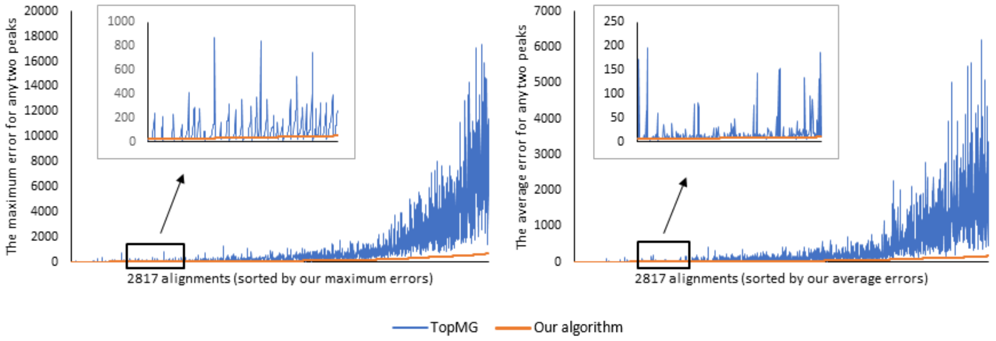

Fig. S6. MME and AME comparison curves between TopMG and TopMGFast for 2817 alignments when using the peak-dependent error tolerance. The 2817 alignment has been sorted by MMEs and AMEs of TopMGFast in non-decreasing order.

unreasonable mass error indicates that the alignment is not reliable. Of course, there still exist some alignments that the MMEs reported by TopMG are smaller than those of TopMGFast. But the MMEs of TopMGFast under those cases are still reasonable and less than $2\delta = 54$. Most AMEs reported by TopMG are much larger than those of TopMGFast.

B. The MME and AME values for all 2817 spectra when using the peak-dependent error tolerance

Fig. S6 illustrates the MMEs and AMEs for the 2817 spectra when we use the peak-dependent error tolerance. As shown in Fig. S6, there is an upper bounder 2δ for the MMEs reported by TopMGFast for all the 2817 alignments. Lots of alignment generated by TopMG has huge MMEs from 1500 to 28000. Again, any alignment with a huge mass difference for a pair of matched peaks should be considered as unreliable. This illustrates that TopMGFast can generate more accurate alignment when using peak-dependent error tolerance.
